# Fatty Acid Oxidation Fuels Mitochondrial Respiration to Drive Epidermal Stem Cell Fate and Barrier Regeneration

**DOI:** 10.21203/rs.3.rs-6636875/v1

**Published:** 2025-06-04

**Authors:** Anna Mandinova, Kristina Todorova, Stefano Sol, Fabiana Boncimino, Andrea Clocchiatti, Kazuki Takagaki, Enkhtuul Gantumur, Mihaela Ruseva, Meaghan Cadieux, Michelle Liu, Victor Neel, Amy Colwell, Christine Lian, George Murphy, Peter Carmeliet

**Affiliations:** Massachusetts General Hospital, Harvard Medical School; Massachusetts General Hospital, Harvard Medical School; Massachusetts General Hospital and Harvard Medical School; Massachusetts General Hospital, Harvard Medical School; Massachusetts General Hospital, Harvard Medical School; Massachusetts General Hospital, Harvard Medical School; Massachusetts General Hospital, Harvard Medical School; Massachusetts General Hospital, Harvard Medical School; Massachusetts General Hospital, Harvard Medical School; Massachusetts General Hospital, Harvard Medical School; Massachusetts General Hospital, Harvard Medical School; Massachusetts General Hospital, Harvard Medical School; Brigham and Women’s Hospital, Harvard Medical School; Brigham and Women’s Hospital, Harvard Medical School; VIB Center for Cancer Biology

**Keywords:** Epidermal Stem Cells, Stem Cell Differentiation, Metabolism, Mitochondrial Respiration, Fatty Acid Oxidation

## Abstract

The skin’s barrier function relies on the epidermis, whose structural integrity is maintained by basal stem cells that continuously renew and differentiate to form the multilayered epidermal architecture. Disruptions in epidermal differentiation underlie numerous hyperproliferative and inflammatory skin disorders. While transcriptional and epigenetic mechanisms are known to regulate the late stages of this process, the molecular events driving the early commitment to differentiation remain elusive. Here, we reveal that early mitochondrial reprogramming, characterized by the activation of oxidative phosphorylation (OXPHOS), is a critical determinant of differentiation initiation. Our findings identify fatty acid oxidation (FAO) as the primary metabolic pathway fueling OXPHOS during this process. Pharmacological and genetic inhibition of FAO, both in vitro and in vivo, disrupts differentiation and compromises the regeneration of the epidermal barrier, causing defective responses to physical insults. Mechanistically, FAO enables ATP production in committed epidermal cells to support the high energy demand of the differentiation process, establishing a direct link between lipid metabolism and epidermal homeostasis. These results uncover a previously unrecognized role for metabolic reprogramming in epidermal stem cell fate and highlight FAO as a novel target for therapeutic interventions to restore barrier function in pathological conditions.

The skin, the largest and most intricate organ of the human body, functions as a critical barrier against environmental insults, including UV radiation and pathogens ^1^. The epidermis, its outermost layer, is pivotal to this barrier function and is maintained through regeneration via differentiation and maturation of approximately every four weeks, driven by epidermal stem cells (ESC) along the basement membrane ^2,3^. These cells continuously divide and differentiate into specialized keratinocytes ^4^, replenishing the basal layer while forming the protective stratum corneum ^1,5,6^.

Recent advances in lineage tracing and genetic manipulation have revealed a dynamic model of epidermal homeostasis, highlighting ESC’ binary decisions to either divide or commit to differentiation ^6–8^. Disruptions in this process underlie skin disorders such as psoriasis, atopic dermatitis, and carcinomas, collectively imposing a significant healthcare burden—estimated at $75 billion annually in the U.S. ^9^. Understanding the molecular mechanisms governing epidermal renewal and differentiation is essential for developing effective therapies for these conditions. Transcription modifiers such as p63 ^10,11,12^, Notch ^13–16^, and members of the AP-1 (e.g., c-Jun, JunB) ^17–19^ and AP-2 ^20^ family play central roles in driving gene expression programs associated with differentiation, including the induction of differentiation markers such as keratin 1 (KRT1), keratin 10 (KRT10), involucrin, filaggrin and loricrin ^21–24^ and simultaneous suppression of stem cells markers such as p63 and members of the Integrin family of proteins ^25^. The activity of these transcriptional regulators is intricately modulated by epigenetic mechanisms, such as histone modifications ^26^, DNA methylation ^27^, and chromatin remodeling, which enable precise spatial and temporal control of gene expression during differentiation ^28–30^.

Additionally, non-coding RNAs such as microRNAs (e.g., miR-203) ^31^, RNA modifications and RNA binding proteins (e.g., YBX1) act as post-transcriptional regulators ^32^, facilitating keratinocyte transition to a differentiated state ^33^. The epidermis is a metabolically active organ that maintains internal homeostasis by integrating external signals into biological processes ^34^. Mitochondria serve as central mediators of epidermal cellular metabolism. They are the primary source of reactive oxygen species (ROS), which regulate key cellular functions, including epidermal differentiation, immune responses, and stress adaptation ^35^. Additionally, mitochondrial calcium uptake is essential in keratinocyte differentiation, as its inhibition disrupts epidermal differentiation ^36^. Interestingly, in in vitro models of human ESC differentiation, the transcriptional and epigenetic changes become detectable approximately 48 hours after initiating the differentiation process. However, this observation leaves unanswered a critical and highly relevant question: what regulates the onset of differentiation? Understanding the upstream signals and regulatory cues that trigger this key process for skin homeostasis remains a major challenge in the field.

In this study, we investigated the early initiation of the differentiation program. We discovered that before the onset of the transcriptional changes that characterize epidermal differentiation, ESC undergo a comprehensive metabolic reprogramming, marked by a shift to mitochondrial respiration. This metabolic transition is not a consequence of the upward movement of committed cells and their immediate exposure to the air surface; instead, it occurs at an early commitment stage. The switch to OXPHOS is essential for initiating epidermal differentiation, preceding previously characterized transcriptional and epigenetic changes. Mitochondrial respiration increases ATP production, which is likely required to meet the high energy demands of the differentiation process. Furthermore, we identified FAO as the exclusive metabolic pathway fueling this shift, underscoring its crucial role in epidermal differentiation.

Our findings reveal a previously unrecognized mechanism that triggers regulates epidermal homeostasis. They also highlight the importance of mitochondrial ATP production and FAO as key determinants of epidermal barrier maintenance.

Environmental interactions lead to the daily desquamation of millions of dead cells from the superficial layers of the human epidermis, potentially resulting in structural disruptions to the epidermal surface. The epidermal barrier is subsequently replenished by the committing-to-differentiation progeny of basal ESC, which progressively migrate away from the basement membrane toward the upper differentiated layers. This upward migration is counterbalanced by the proliferation of ESC, ensuring the continuous renewal of the basal layer.

In vitro modeling of epidermal homeostasis relies on primary cultures of human ESC, which proliferate through two distinct modes: “balanced” and “expanding” ^37^. Under low confluency conditions, proliferating ESC predominantly adopt an “expanding mode,” mirroring their self-renewal behavior during homeostasis and in response to injury in vivo. This results in a relatively homogeneous population that can be sustained across multiple cellular generations. As cells reach confluence, the “expanding mode” transitions into the “balanced mode”, promoting commitment to differentiation and leading to the formation of a heterogeneous population—an essential process for the development of stratified epithelium ^32,38,39^. It is now well established that the differentiation program in vitro can be initiated upon the addition of exogenous calcium, which induces the transcription and expression of various differentiation markers (Figure 1A).

A hallmark of ESC differentiation is the loss of colony-forming capacity. Using an in vitro model of epidermal homeostasis, we demonstrate that calcium-stimulated ESC rapidly commit to differentiation and lose their colony-forming ability well before the detectable expression of early differentiation markers such as KRT10 (Figure 1B–D). Primary ESC cultures were derived from non-sun-exposed areas of the human body to minimize the influence of chronic UV-induced cellular damage. Previous studies utilizing the same in vitro human model have established a 48–72-hour timeline for the complete execution of the transcriptional differentiation program. Consistent with these findings, we observed an increase in differentiation markers (KRT10, KRT1, and Involucrin) at 24 hours post-stimulation. In contrast, suppression of key stem cell markers, such as p63, KRT5, and ITGA6, was not yet detectable (Figure 1E).

We performed RNA sequencing to investigate the transcriptional landscape during the earliest phases of differentiation commitment. ESC collected immediately before calcium addition served as controls. Surprisingly, despite the early loss of the stem cell phenotype and a marked decrease in colony-forming ability, we observed only minimal changes in the transcriptional profile of ESC at 2- and 6-hours post-stimulation (Figure 1F–H). Substantial transcriptional alterations became evident only at 24 hours post-stimulation (Figure 1I). Differential gene expression analysis revealed strong enrichment for pathways associated with epidermal development and epithelial cell differentiation, with “Cornified Envelope” emerging as the most significantly enriched Cellular Component (Figure 1J).

Although this analysis used primary ESC cultures derived from female donors, a parallel experiment with male donors confirmed these findings (Figure S1A, B), demonstrating no gender bias in the experimental conditions. Collectively, our data surprisingly indicate that commitment to differentiation and the loss of the stem cell phenotype occur before the well-characterized transcriptional changes traditionally used to define the process of ESC differentiation.

Stem cell fate decisions and lineage commitment are highly energy-demanding processes that necessitate profound changes in intracellular metabolism. Given that ESC commitment to differentiation is associated with a drastic increase in intracellular calcium levels in vivo and in vitro, we aimed to elucidate the metabolic changes occurring during the early stages of commitment.

Our RNA sequencing data revealed a lack of transcriptional changes in metabolism-related genes. However, it remains possible that differentiation initiation is governed by post-transcriptional metabolic rewiring. To investigate this, we directly measured real-time metabolic changes in ESC progeny undergoing differentiation. Our data revealed a significant increase in the oxygen consumption rate (OCR), indicative of a shift toward oxidative phosphorylation (OXPHOS), as early as 2 hours post-calcium induction. This metabolic shift, also characterized by a significantly elevated basal and maximal respiration, persists throughout the early stages of differentiation and up to 24 hours post-stimulation (Figure 2A and S2A).

Because exogenous calcium is known to activate mitochondrial metabolism and enhance OXPHOS artificially ^40^, we sought to determine whether OCR upregulation is an intrinsic feature of ESC differentiation. Indeed, this metabolic shift was recapitulated in differentiating ESC progeny upon suspension culture, independent of calcium addition (Figure S2B), confirming that metabolic reprogramming is a fundamental aspect of early ESC differentiation.

Given the absence of transcriptional changes in genes regulating mitochondrial function, we hypothesized that differentiation-committed progeny regulate OXPHOS through mitochondrial dynamics, as previously suggested ^41,42^. To assess the correlation between increased mitochondrial respiration and mitochondrial architecture, we visualized and quantified mitochondrial network changes during differentiation (Figure 2B–E and S2C-E). Our analysis revealed profound morphological remodeling, including an increase in the number of networks coupled with fewer and shortened branches. Taken together, the change in these parameters implies active mitochondrial restructuring and fragmentation during commitment to differentiation ^43,44^.

To further characterize metabolic reprogramming, we performed an untargeted metabolomic analysis of control ESC and calcium-initiated progeny using liquid chromatography-tandem mass spectrometry (LC-MS/MS). Initiation of differentiation led to significant changes in the levels of several metabolites (Figure 2F and S2F, G). Consistent with the metabolic shift toward OXPHOS, metabolites involved in the Citric Acid Cycle and Fatty Acid Oxidation were upregulated, while glycolytic intermediates were downregulated (Figure 2G–H). Notably, we also observed an increase in Acetylcarnitine, and Thiamine Phosphate, while D-glyceraldehyde 3-phosphate was reduced (Figure S2H).

These findings substantiate our mitochondrial flux analysis and morphological visualization, demonstrating that differentiating keratinocytes undergo profound metabolic reprogramming toward OXPHOS well before the onset of transcriptional changes.

OXPHOS, as the final step in nutrient oxidation, integrates the metabolism of glucose, fatty acids, and amino acids. Although cellular substrate preference mechanisms remain incompletely understood, recent studies suggest that different tissues exhibit distinct metabolic dependencies ^45^ highlighting the critical role of various metabolic substrates in regulating fundamental cellular functions, including differentiation.

To identify the primary substrate driving mitochondrial activation and fueling ESC differentiation, we investigated the metabolic inputs sustaining OXPHOS. Specifically, we inhibited the following key metabolic pathways: glutamine oxidation via glutaminase using bis-2-(5-phenylacetamido-1,3,4-thiazol-2-yl)ethyl sulfide (BPTES) ^46^, pyruvate flux through the mitochondrial pyruvate carrier (MPC) using the α-cyanocinnamate analog UK5099 ^47^, and fatty acid oxidation (FAO) through irreversible inhibition of the carnitine palmitoyltransferase-1 (CPT1) via etomoxir ^48^ (Figure 3A).

We focused on the magnitude of changes in baseline and maximal respiration 24 hours after the initiation of differentiation. Inhibition of these three mitochondrial substrate oxidation pathways reduced baseline respiration in control ESC, suggesting utilization of all three substrates and, to some extent, plasticity in mitochondrial metabolism in proliferating ESC. However, while BPTES and UK5099 did not prevent the increase in baseline and maximal respiration during the early phases of differentiation, inhibition of FAO via etomoxir completely abolished mitochondrial reprogramming in differentiating keratinocytes (Figure 3B–D and S3A).

To assess whether reduced FAO through etomoxir directly impacts ESC’ commitment to differentiation, we measured the expression of KRT10 24 hours after calcium initiation. We observed that inhibiting the transport of long-chain fatty acids into the mitochondria via CPT1 completely prevented ESC from committing to differentiation (Figure 3E). In contrast, when oxidation of other mitochondrial substrates, such as pyruvate and amino acids, was pharmacologically inhibited, ESC were still able to initiate differentiation and express KRT10 at 24 hours post-initiation (Figure 3E). Given that etomoxir has been shown to exhibit a variety of off-target effects ^49^, we also suppressed mitochondrial FAO by inhibiting CPT1 expression through siRNA-mediated knockdown. This approach led to a reduction in mitochondrial reprogramming during keratinocyte commitment to differentiation. Subsequently, it inhibited the differentiation program, confirming the essential role of mitochondrial FAO in ESC differentiation (Figure S3B, C). Treatment with etomoxir also rescued the colony-forming ability of ESC even after 24 hours of incubation with calcium, indicative of preserving the stem cell phenotype while lacking the ability to initiate differentiation (Figure 3F, G).

While animal models only partially recapitulate human epidermal homeostasis due to significant differences in organ structure, human skin equivalents serve as a well-characterized and widely used model for studying ESC renewal and commitment to differentiation. In this study, we utilized full-thickness 3D skin equivalents composed of both dermal and epidermal components to investigate the consequences of reduced mitochondrial FAO on epidermal homeostasis and differentiation. Etomoxir treatment resulted in defective epidermal formation, with loss of keratohyalin granules and visible retention of nucleated cells in the upper epidermal layer, suggesting severe differentiation defects (Figure 3H–J). Immunostaining for differentiation markers in the equivalents revealed significantly reduced expression of the early differentiation marker KRT10 (Figure S3D, E).

To further explore the in vivo relevance of mitochondrial FAO for epidermal homeostasis, we employed a genetic mouse model with epidermis-specific deletion of CPT1 ^50^, driven by the Keratin 14 promoter (K14Cre; CPT1A^f/f^) (Figure 3K, L). Notably, the CPT1 deletion did not result in major phenotypic alterations in the skin of neonatal and adult mice under baseline conditions (Figure 3M and S3F). This outcome was anticipated, as CPT1 functions in conjunction with its partner CPT2 and CACT (carnitine-acylcarnitine translocase) to facilitate the transport of fatty acids into the mitochondria, with some degree of functional redundancy. Epidermal differentiation is the primary driver of epidermal homeostasis and triggers the self-renewal of ESC in the basal layer to compensate for differentiated progeny ^7^. Therefore, we induced ectopic epidermal differentiation in control CPT1A^f/f^ and K14Cre; CPT1A^f/f^ mice by tape stripping and removing the corneocytes from the outermost epidermal layers ^51^. It has been shown that this insult to the epidermal barrier and subsequent differentiation results in the excessive division of ESC that aims to replenish the basal layer and balance the loss of the differentiating progeny ^7,52^. While control CPT1A^f/f^ mice exhibited aberrant proliferation in the basal layer following tape stripping, K14Cre; CPT1A^f/f^ mice failed to respond, indicating a deficiency in their ability to initiate detachment from the basal membrane, upward movement and compensatory proliferation (Figure 3M, N). This suggests that mitochondrial FAO is essential for the stress-induced initiation of epidermal differentiation. To further confirm that ectopic differentiation is indeed impaired upon FAO blockade, we tested if tape stripping would interfere with the very early commitment of ESC progeny to differentiation. In the mouse epidermis, KRT10 is the first upregulated differentiation marker, and early molecular changes associated with differentiation are characterized by an increase in KRT10 levels within a restricted population of basal keratinocytes, which still express high levels of the stem cell markers KRT14 and KRT15 ^8^. To investigate the effects of deficient mitochondrial FAO on these early differentiation events, we focused on KRT10/KRT15 double-positive basal epidermal cells in response to tape stripping. As expected, control CPT1A^f/f^ mice exhibited a substantial increase in the number of early differentiating double-positive cells, whereas K14Cre; CPT1A^f/f^ mice failed to show a similar response (Figure 3S G, H), indicating a blockade of ESC differentiation.

Taken together, our findings demonstrate that mitochondrial FAO is specifically required for the early commitment to epidermal differentiation.

FAO integrates several metabolic pathways, with acetyl-CoA as its primary end product. Acetyl-CoA is an essential intermediate involved in various cellular processes. The levels of acetyl-CoA in both the nucleus and cytoplasm have been shown to influence the epigenetic regulation of gene expression across different cell types, including epidermal keratinocytes ^53^. To explore the cellular mechanisms by which FAO supports ESC’ differentiation, we first examined whether FAO promotes epidermal differentiation by maintaining the cytoplasmic acetyl-CoA pool ^54^. Differentiation in ESC cultures was induced with calcium, in the presence or absence of etomoxir. We then assessed whether acetyl-CoA precursors such as acetate and citrate could bypass FAO inhibition to sustain epidermal differentiation. Treatment with acetate or citrate failed to restore epithelial differentiation suppressed by etomoxir, as indicated by the lack of KRT10 expression (Figure 4A). This indicates that the effects of suppressed FAO on ESC are not a consequence of limited levels of cytoplasmic acetyl-CoA.

Given the constant exposure of the human epidermis to air and the high efficiency of ATP production via FAO and OXPHOS when oxygen is abundant, we also explored whether FAO primarily supports ATP production. Mitochondrial flux analysis revealed a significant increase in ATP levels during differentiation (Figure 4B), an effect blocked by etomoxir treatment (Figure S4A). To investigate this further, we conducted a series of perturbations targeting the cellular machinery responsible for ATP production (Figure 4C). In normal mammalian cells, ATP is derived from both glycolysis and mitochondrial OXPHOS. To block mitochondrial ATP production, we treated calcium-induced ESC with oligomycin and antimycin, which inhibit the electron transport chain and ATP synthase within the mitochondria ^55^. This treatment completely blocked epidermal differentiation, as assessed by KRT10 expression (Figure 4D). Similar results were obtained with arsenite, a compound that disrupts key enzymes in the electron transport chain through its interaction with thiol groups, thereby reducing ATP production via OXPHOS ^56^.

Although glycolysis contributes to a smaller portion of ATP production, we also evaluated its role in epidermal differentiation. Inhibition of glycolytic ATP production with 2-deoxyglucose (2-DG), a glucose analog (Figure 4D), did not prevent ESC from committing to differentiation, highlighting that mitochondrial but not glycolytic ATP production is essential for epidermal homeostasis. To further confirm these findings, we used Piericidin A, an inhibitor of mitochondrial Complex I, to treat full-thickness 3D skin equivalents. This intervention resulted in defects similar to those observed with FAO inhibition by etomoxir: impaired epidermal formation, a thinner multilayered structure, retention of nucleated cells in the upper epidermal layers, and failure to induce differentiation markers such as KRT10 (Figure 4E–I).

Modeling epidermal differentiation in intact human skin presents challenges. However, the replenishment of the basal layer through ESC division can be studied in intact skin. A discrete population of basal ESC continually proliferates to replace differentiated progeny during barrier renewal. In human skin, these proliferating ESC constitute approximately 8-10% of the basal layer and are positive for the proliferation marker Ki67. We hypothesized that mitochondrial activation would subtly enhance homeostatic differentiation and lead to an increased number of Ki67-positive basal keratinocytes. Mitochondrial NAD+ (nicotinamide adenine dinucleotide) is critical for ATP generation as it acts as an electron carrier in the electron transport chain. The reduction of NAD+ to NADH during the citric acid cycle creates a proton gradient across the mitochondrial membrane, which drives ATP synthesis via OXPHOS ^57^. Treatment of patient-derived human skin explants with the NAD+ precursor NMN (nicotinamide mononucleotide) resulted in a significant increase in Ki67-positive cells in the basal layer, indicating activation of epidermal turnover (Figure S4B, C).

In summary, these data indicate that FAO is not required to supply acetyl-CoA for histone acetylation of differentiation-related genes during basal epidermal keratinocyte differentiation. However, it is indispensable for ATP production, which is essential for executing the differentiation program. In this context, inhibiting mitochondrial ATP production by blocking FAO disrupts ESC differentiation and impedes replenishing the epidermal barrier.

Our study demonstrates that homeostatic epidermal renewal, driven by ESC differentiation, necessitates metabolic reprogramming towards OXPHOS at the early stages of commitment. Furthermore, we establish that the activation of mitochondrial respiration and ATP production is critically dependent on fatty acid β-oxidation. Using genetic loss-of-function experiments and pharmacological inhibition of FAO both in vitro and in vivo, we show that blocking this metabolic pathway disrupts ESC differentiation, thereby impairing epidermal renewal and compromising barrier function.

Defective epidermal differentiation is increasingly recognized as a key molecular driver of various skin pathologies, including inflammatory skin disorders, benign hyperplastic lesions, and ichthyosis ^58–60^. It is widely accepted that epidermal differentiation is governed by a tightly controlled transcriptional program, regulated at the epigenetic level, which promotes the expression of pro-differentiation factors while simultaneously suppressing stem cell-associated genes ^3,7,24,25^. However, the precise molecular mechanisms that initiate this complex process remain elusive. A well-established factor in ESC commitment is the increase in intracellular calcium levels, yet the molecular events linking this calcium spike to the activation of the differentiation-associated transcriptional response remain poorly understood ^9^. Building the multilayered structure of the epidermis is an energy-intensive process, requiring high ATP levels and an adequate supply of biomolecular precursors. Cellular metabolism plays a pivotal role in meeting these demands, and previous studies have implicated increased ROS levels, amino acids, and lipids as key drivers of epidermal differentiation ^35^. To elucidate the role of metabolism in epidermal homeostasis, we employed a well-established in vitro ESC differentiation model in combination with 3D skin equivalents. Our findings reveal that metabolic reprogramming towards mitochondrial respiration occurs very early after calcium stimulation and is essential for executing the differentiation program. This shift to OXPHOS is likely sustained by mitochondrial calcium uptake and the availability of oxygen. Recently, mitochondrial deficiencies in ESC have been implicated in the pathogenesis of common epidermal disorders, such as atopic dermatitis, independently of immune system involvement ^61^.

Moreover, we demonstrate that differentiating ESC rely exclusively on FAO to sustain mitochondrial respiration and ATP production. These findings were corroborated in skin organoid models as well as in FAO-deficient mouse models, reinforcing the critical role of FAO in epidermal differentiation. The preferential engagement of FAO is likely driven by the availability of lipids within the epidermal environment.

Our study identifies fundamental metabolic determinants of early epidermal differentiation and raises several important questions for future research. What role does FAO play in defective differentiation responses at the onset of inflammatory skin reactions? Could targeting FAO serve as a potential therapeutic strategy for treating epidermal differentiation disorders? Addressing these questions will provide deeper insights into epidermal biology and may inform novel approaches for managing skin diseases characterized by impaired differentiation.

## Methods

### Animal Studies

CPT1A fl/fl mice were provided from Laboratory animal center KU Leuven, Belgium and K14-Cre mice B6N.Cg-Tg(KRT14-cre)1Amc/J were purchased from The Jackson Laboratory. Mousegenotyping for CPT1A fl/fl mice was performed by PCR starting from mouse tail DNA isolated with DNeasy Blood & Tissue Kit (Qiagen 69504). For CPT1A, the following oligonucleotides primers were used:

CPT F: 5’ CAG-CTG-CTC-CAC-ACC-AAG-GCT 3’

CPT R: 5’ TGC-CCT-TCT-ACT-GTC-ACA-TGG 3’

Results: loxlox band: 403 bp; Wt band: 290 bp

Samples (mice tail) for K14-Cre genotyping were sent to Transnetyx Inc.

For mice back skin tape strip, 7-week-old mice were used to affirm resting of the hair follicles due to telogen phase of their hair cycle. The mice were anesthetized with isoflurane and the back skin was shaved and epilated. The tape stripping, using Tegaderm film, was performed the day after hair removal. To standardize the degree of epidermal disruption elicited by tape stripping, the trans epidermal water loss (TEWL) is measured using a Dermalab TEWL probe (CortexTechnology, Denmark). All animal experimental protocols were approved by IACUC of Massachusetts General Hospital.

### Cell Culture

Healthy skin samples taken during surgical operations were used to isolate human primary keratinocytes and dermal fibroblast. Following the removal of subcutaneous adipose tissue from the human skin specimens, the tissue pieces were incubated in dispase solution (StemCell Technologies) at 4°C overnight. After digestion, the epidermis and the dermis were separated, chopped into small pieces and processed separately.

The dermis was incubated with Collagenase/Hyaluronidase (StemCell Technologies) 1X at 37°C overnight. Fibroblast were then passed through a 70 μm cell strainer and cultured in in DMEM (Gibco) supplemented with 10% FBS and 1% antibiotic/antimycotic and maintained at 37 °C in 5% CO_2_.

The epidermis was incubated in 0.05% Trypsin-EDTA (Gibco) for 10 minutes at 37°C. The human primary keratinocytes were then passed through a 70 μm cell strainer, seeded in a precoated dish, and cultured in Keratinocyte SFM (K-SFM, Thermo Fisher Scientific) supplemented with 0.2 ng/mL human recombinant epidermal growth factor (EGF), 30 μg/mL bovine pituitary extract (BPE), and 1% antibiotic/antimycotic. Cultures were maintained at 37 °C in 5% CO_2_. Cells were cultured in K-SFM supplemented with 30ug/ml BPE and 0.3ng/ml EGF with 5% CO_2_ at 37°C.

For differentiation, keratinocytes at a confluency of 50–60% were treated with 1.5 mM CaCl_2_ (C1016, Sigma-Aldrich) for 2, 6 or 24 hours. Differentiation of keratinocytes upon suspension culture was performed as described previously (reference). Briefly, 500000 cells of keratinocytes were resuspended in 1.45% methyl cellulose in complete medium and seeded in 12-well with a super low cell attachment surface (Nunclon Sphera Plates, Thermo Scientific). Cells were incubated at 37°C in 5% CO_2_ and harvested at 24 hours by diluting the methylcellulose with PBS. Cells were collected and subjected to multiple PBS washes by centrifugation.

Swiss3T3 were cultured in DMEM (Gibco) supplemented with 10% FBS and 1% antibiotic/antimycotic and maintained at 37 °C in 5% CO_2_.

### Skin explant

7mm punch biopsy skin explants were placed dermal side down onto membranes of trans well inserts (Costar) in contact with growth medium DMEM (Gibco), containing 10% human serum (Gemini Bio Products) and 1% antibiotic/antimycotic. The surface of the biopsy was kept in contact of the air. The medium in the bottom part of the well was supplemented with DMSO or the indicated concentration of NMN. The so treated skin explants were harvested after 24 hours and fixed in 10% formalin overnight for paraffin sections.

### Full-thickness 3D skin equivalents

Dermal fibroblasts were mixed with collagen matrix and seeded in deep wells (BD Biosciences). Human keratinocytes were placed over the dermal equivalent and grown submersed in KGM-2 Bullet Kit (Lonza) for 48 hours. 3D skin cultures were subjected to air–liquid interface (ALI) in differentiation media (KGM Bullet Kit, Lonza, with 25mg of ascorbic acid, 10 μg/mL of transferrin, and 1M of CaCl_2_) for 7 days. Starting on the day of establishment of ALI, 3D skin cultures were treated with the indicated concentration of Etomoxir or Piericidin A.

### siRNAs and transfection methods

Predesigned siRNAs for human CPT1A and control siRNAs were purchased from Dharmacon (Cat. No: L-009749-00-0010, D-001206-13-05). Cells were transiently transfected using HiPerFect Transfection Reagent (Qiagen).

### Colony forming assays

Swiss3T3 cells were treated with 4 μg/ml mitomycin C for 3 hours and plated in 6-well plates 24 hours prior seeding the keratinocytes. Human primary keratinocytes were treated with 1.5 mM CaCl_2_ and 40 μM Etomoxir for the indicated time. After that, 100 cells of treated keratinocytes were seeded in 6-well plates containing the layer of Swiss3T3 feeder cells. Clonal density cultures were maintained in FAD medium (50% DMEM, 50% DMEM/F12 with the addition of 5% FBS, 5μg/ml insulin, 0.18 mM adenine, 0.5μg/ml hydrocortisone, 1 × 10^−9^M cholera toxin and 10ng/ml EGF) for 10 days, fixed with 4% PFA and stained with 1% crystal violet.

### SDS-PAGE and Western blot

Cells were lysed in RIPA buffer (Boston Bio Products). Samples were prepared after mixing 3 parts of the clear lysates with 1 part of 4x NuPAGE LDS Sample Buffer (Invitrogen) and b-mercaptoetanol (EMD Millipore). Samples were boiled and loaded on denaturing SDS-PAGE gels followed by western blotting. Membranes were blocked with 5% nonfat-dry milk and incubated with primary antibodies overnight at 4°C. The primary antibodies used for Western blot analysis were: anti-vinculin (sc73614 - Santa Cruz), anti-KRT10 (ab76318, Abcam), anti-CPT1A (15184-1-Ap, Proteintech). Membranes were incubated for 1h at RT with the appropriate horseradish peroxidase-conjugated secondary antibody and detected by chemiluminescence.

### RNA Isolation and RT-qPCR

Total RNA was extracted from cells using RNeasy Mini Kit (Qiagen) and retro-transcribed to cDNA using Maxima First Strand cDNA Synthesis Kit (Thermo Fisher Scientific). RT-qPCR was performed using SYBR green Master Mix (Applied Biosystems) in a Quant Studio 3 instrument (Applied Biosystems). Target genes were quantified using the following specific oligonucleotide primers and normalized to human 36B4 expression.

**Table:** 

Gene	Forward primer (5’-3’)	Reverse primer (5’-3’)
36B4	TGGTCATCCAGCAGGTGTTCGA	ACAGACACTGGCAACATTGCGG
KRT10	CCTGCTTCAGATCGACAATGCC	ATCTCCAGGTCAGCCTTGGTCA
KRT1	CAGCATCATTGCTGAGGTCAAGG	CATGTCTGCCAGCAGTGATCTG
INV	GGTCCAAGACATTCAACCAGCC	TCTGGACACTGCGGGTGGTTAT
TP63	CAGGAAGACAGAGTGTGCTGGT	AATTGGACGGCGGTTCATCCCT
KRT5	GCTGCCTACATGAACAAGGTGG	ATGGAGAGGACCACTGAGGTGT
ITGA6	CGAAACCAAGGTTCTGAGCCCA	CTTGGATCTCCACTGAGGCAGT

### Mitochondrial function measuring (Seahorse XF Assay)

Oxygen consumption rate (OCR) was measured using XF Cell Mito Stress Test Kit (103015-100) on Agilent Seahorse XFe24 Analyzer. The utility plate was collagen coated and 15000 cells/well were plated (except in the background wells) in 100 μl Keratinocyte-SFM medium. After 1h incubation for evenly cells distribution, 150 μl of growth medium are added to each well. Depends on the experiment, cells were treated with the indicated concentration of mitochondria pathways inhibitors and CaCl_2_. Seahorse sensor cartridge was incubated with Seahorse Calibrant solution overnight at 37°C in CO_2_ free incubator. Cells in the utility plate were first washed and then incubated with fresh 500 μl assay medium (Seahorse media XF DMEM medium pH 7.4, cat #103575-100, supplemented with 10 mM glucose, 1 mM pyruvate to final and 2 mM glutamine) for 1h at 37°C in CO_2_ free incubator prior the assay. OCR was monitored at baseline and throughout sequential injections of oligomycin (1 μM), carbonyl cyanide-4-(trifluoromethoxy)phenylhydrazone (FCCP) (1 μM) and rotenone/antimycin A (0.5 μM each).For keratinocytes differentiated upon suspension culture, 40000 already differentiated cells were resuspended in 500 μl of fresh assay medium and plated in seahorse utility plate coated in advance with Poly-D-Lysine. After gentle spin to let them attach, incubate for 1h at 37°C in CO_2_ free incubator prior the assay.

### Mitotracker

Human primary keratinocytes were incubated for 30 min with 100 nM MitoTracker Red CMXROS (Invitrogen). Then, cells were fixed for 15 min with 4% paraformaldehyde. Fluorescence images were acquired using a confocal microscope (Nikon). An average of 20 cells per condition from 3 independent experiments were used to perform the Mitochondrial Network Analysis (MiNA) with ImageJ software.

### Metabolomics LC-MS sample preparation

Cells were lysed in pre-chilled 80% Methanol HPLC Grade. After centrifuging for 10 min at 10000 g, the supernatant was transferred into new tube and dried in the Speed-Vac concentrator. Dried samples were resuspended using 20 uL HPLC grade water for mass spectrometry.5-7 μL were injected and analyzed using a hybrid 6500 QTRAP triple quadrupole mass spectrometer (AB/SCIEX) coupled to a Prominence UFLC HPLC system (Shimadzu) via selected reaction monitoring (SRM) of a total of 298 endogenous water-soluble metabolites for steady-state analyses of samples. Some metabolites were targeted in both positive and negative ion mode for a total of 309 SRM transitions using positive/negative ion polarity switching. ESI voltage was +4950V in positive ion mode and −4500V in negative ion mode. The dwell time was 3 ms per SRM transition and the total cycle time was 1.55 seconds. Approximately 9-12 data points were acquired per detected metabolite. Samples were delivered to the mass spectrometer via hydrophilic interaction chromatography (HILIC) using a 4.6 mm i.d x 10 cm Amide XBridge column (Waters) at 400 μL/min. Gradients were run starting from 85% buffer B (HPLC grade acetonitrile) to 42% B from 0-5 minutes; 42% B to 0% B from 5-16 minutes; 0% B was held from 16-24 minutes; 0% B to 85% B from 24-25 minutes; 85% B was held for 7 minutes to re-equilibrate the column. Buffer A was comprised of 20 mM ammonium hydroxide/20 mM ammonium acetate (pH=9.0) in 95:5 water:acetonitrile. Peak areas from the total ion current for each metabolite SRM transition were integrated using MultiQuant v3.0 software (AB/SCIEX).

### Histology and immunofluorescence.

Skin explant, 3D skin cultures and mouse skin were harvested and fixed in 10% formalin overnight for paraffin sections. Paraffin sections were used for Hematoxylin and Eosin (H&E) staining and histological analysis. After standard deparaffinization, 15 minutes permeabilization with 0.5% Triton-X 100 in PBS and heat-induced antigen retriever with citrate-based antigen unmasking solution (Sigma), sections were incubated overnight at 4°C with the following antibodies: anti-KRT5 (03-GP-CK5, ARP); anti-KRT10 (905404, Bio-Legend); Ki67 (ab16667, Abcam). Secondary Alexa Fluor antibodies were incubated for 1 h at room temperature. Nuclei were stained with 1ug/ml Hoechst 33342 (Invitrogen).

### QUANTIFICATION AND STATISTICAL ANALYSIS

#### RNA-seq and expression analysis

RNA library preparation, sequencing, and analysis were conducted at Azenta Life Sciences (South Plainfield, NJ, USA) as follows: Library Preparation with PolyA selection and Illumina Sequencing RNA samples were quantified using a Qubit 2.0 Fluorometer (Life Technologies), and RNA integrity was checked using an Agilent TapeStation 4200 (Agilent Technologies). RNA sequencing libraries were prepared using the NEBNext Ultra II RNA Library Prep Kit for Illumina using the manufacturer’s instructions (NEB). The sequencing library was validated on the Agilent TapeStation (Agilent Technologies), and quantified by using Qubit 2.0 Fluorometer (Invitrogen) as well as by quantitative PCR (KAPA Biosystems). The sequencing libraries were clustered on a flow cell. After clustering, the flow cell was loaded on the Illumina NovaSeq instrument according to the manufacturer’s instructions. The samples were sequenced using a 2x150bp Paired-End (PE) configuration, targeting 30 M reads/sample. Differential expression analysis was performed, and the Wald test was used to generate p-values and Log2 fold changes. Genes with p-values < 0.05 and absolute log2 fold changes ≥ 1 were called as differentially expressed genes for each comparison. A gene ontology analysis was performed on the statistically significant set of genes by Enrichr software. The human GO list was used to cluster the set of genes based on their biological process, cellular component, and determine their statistical significance.

#### Metabolomics LC-MS analysis

Relative metabolite levels were quantified from peak area and normalized to the cells number.

Heatmaps, principal component analysis, volcano plot and enrichment analysis were generated using MetaboAnalyst.

#### Statistical analysis

All datasets derive from at least three independent experiments unless otherwise indicated. Data are presented as the mean of independent experiments ±SD as indicated. All statistical analyses were performed using GraphPad Prism software (version 10.0). In experiments comparing two samples, paired or unpaired, two-tailed t-test was performed, whereas when comparing multiple independent samples, one-way analysis of variance (ANOVA) was performed as described in the legends.

## Figures and Tables

**Figure 1:**
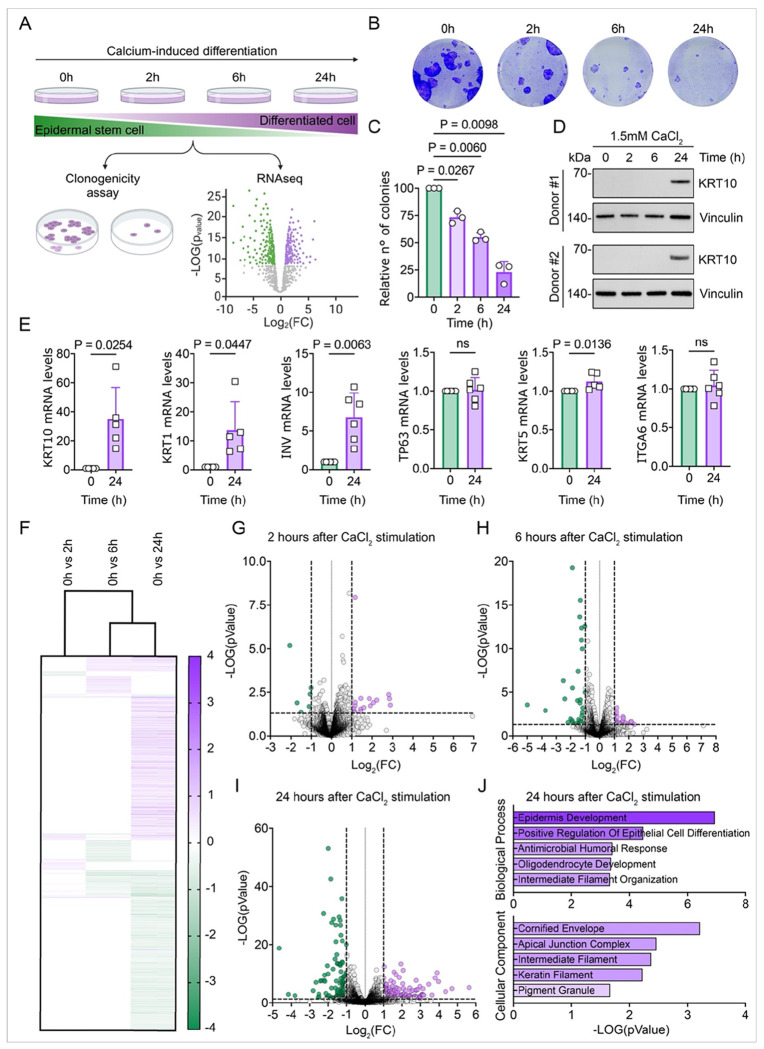
Early differentiation commitment in human ESC happens without significant transcriptional changes (A) Schematic of the experimental design. (B–C) Colony-forming capacity of ESC under basal or differentiated conditions at various time points (0, 2, 6, and 24 hours after calcium addition). Cells were fixed and stained with Crystal Violet. Representative wells are shown in (B), with the corresponding quantification of colony numbers in (C). *n* = 3 human skin donors; *n* = 3 wells per condition per donor. Data are presented as mean ± SD. Statistical analysis was performed using one-way ANOVA followed by Dunnett’s multiple comparisons test. (D) Western blot analysis of KRT10 expression in ESC under basal or differentiated conditions at 0, 2, 6, and 24 hours after calcium. Vinculin was used as a loading control. (E) Expression levels of KRT10, KRT1, INV, TP63, KRT5, and ITGA6 in ESC under basal or differentiated conditions at 24 hours after calcium addition, *n* ≥ 5 donors. Data are shown as mean ± SD. *p*-values were determined using a paired two-tailed *t*-test. (F) Heatmap of significantly differentially expressed genes (*p* < 0.05) between basal and differentiated conditions in ESC at 0, 2, 6, and 24 hours after calcium addition (n = 3). (G–I) Volcano plots of RNA-seq data showing differentially expressed genes between basal and differentiated conditions at indicated time points. Green dots represent significantly downregulated genes (log_2_fold change ≤ −1, *p* < 0.05), and violet dots represent significantly upregulated genes (log_2_fold change ≥ 1, *p* < 0.05, n = 3 donors). (J) Top 5 Gene Ontology (GO) terms identified for biological processes (upper panel) and cellular components (lower panel) from the indicated comparison.

**Figure 2:**
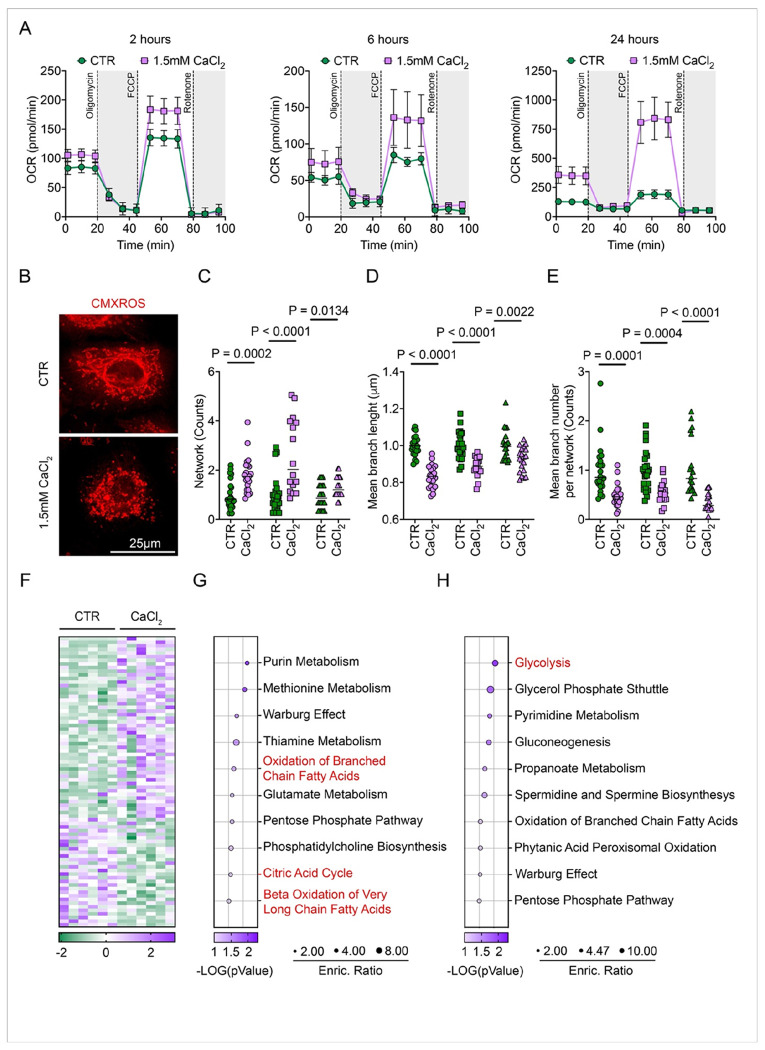
Metabolic reprogramming to OXPHOS promotes the early differentiation commitment of ESCs. (A) OCR analysis of ESC under basal or differentiated conditions at the indicated time points (2, 6, and 24 hours). Cells were sequentially treated with oligomycin, FCCP, and rotenone/antimycin A as indicated (n ≥ 3 donors). Data are presented as mean ± SD. (B) Representative confocal images of mitochondria in ESC under basal or differentiated conditions at 24 hours. Cells were stained with MitoTracker CMXRos (red). Scale bar, 25 μm. (C–E) Mitochondrial network analysis using the MiNA plugin in ImageJ. Quantification of total networks (C), mean branch length (D), and mean branch number per network (E) is shown. n > 15 cells analyzed per donor for each condition. Data are presented as mean ± SD. *p*-values were calculated using an unpaired two-tailed *t*-test. (F) Heatmap of the top 80 differentially expressed metabolites identified by LC-MS-based metabolomics analysis of ESC under basal versus differentiated conditions at 24 hours after calcium addition. Shades of violet and green represent increased and decreased metabolite levels, respectively. (G) Top 10 enriched upregulated metabolic pathways. Circle size indicates the enrichment ratio, and color represents the corresponding *p*-value. (H) Top 10 enriched downregulated metabolic pathways. Circle size indicates the enrichment ratio, and color represents the corresponding *p*-value.

**Figure 3:**
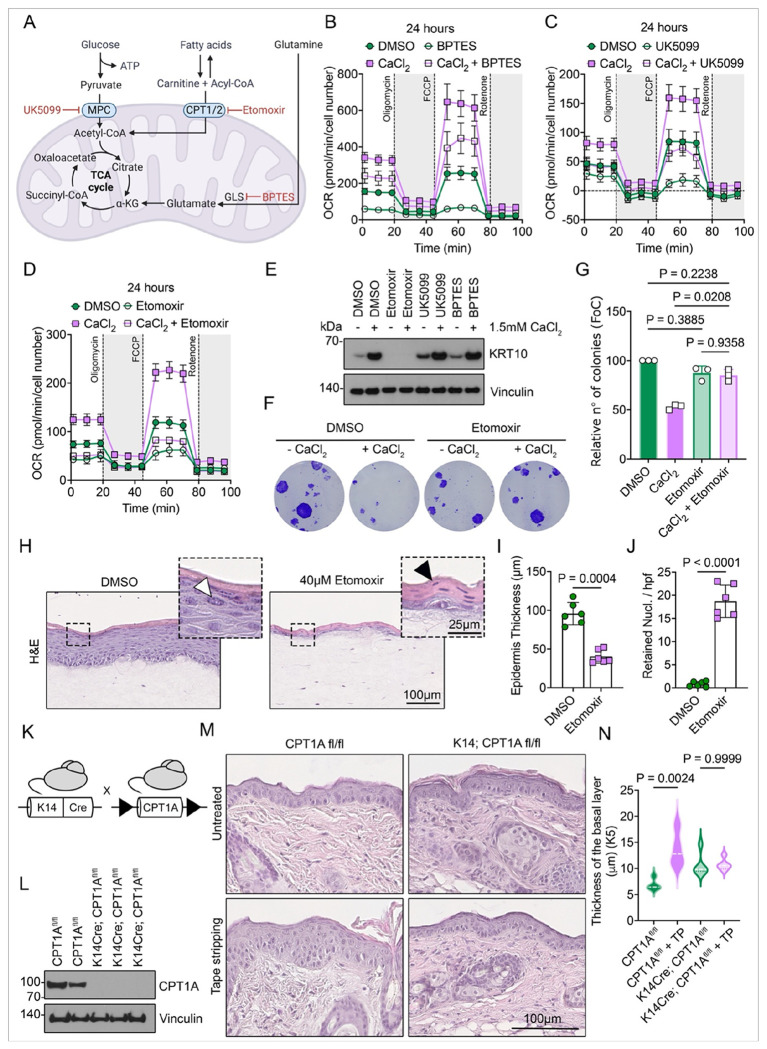
Mitochondrial fatty acid oxidation is crucial for the differentiation of ESCs. (A) Schematic representation of the metabolic pathways and respective interventions. (B–D) OCR analysis of ESC treated for 24 hours with DMSO, 1 μM BPTES, 10 μM UK5099, or 40 μM Etomoxir under basal or differentiated conditions. Cells were sequentially treated with oligomycin, FCCP, and rotenone/antimycin A (n = 2 donors). Data are presented as mean ± SD. (E) Western blot analysis of KRT10 expression in primary human keratinocytes treated with DMSO, 1 μM BPTES, 10 μM UK5099, or 40 μM Etomoxir for 24 hours under basal or differentiated conditions. Vinculin was used as a loading control. (F–G) Colony-forming capacity of ESC treated with DMSO or 40 μM Etomoxir for 24 hours under basal or differentiated conditions. Cells were fixed and stained with Crystal Violet. Representative wells are shown in (F), and quantification of colony numbers is shown in (G). n = 3 donors; n = 3 wells per condition per donor. Data are presented as mean ± SD. Statistical analysis was performed using one-way ANOVA followed by Sidak's multiple comparisons test. (H) Representative low- and high-magnification images of hematoxylin and eosin (H&E) staining of 3D skin cultures treated with DMSO or 40 μM Etomoxir for 7 days. White arrowheads indicate keratohyalin granules and black arrowheads indicate retained nuclei in the cornified layer. Scale bars: 100 μm (low magnification) and 25 μm (high magnification). (I) Quantification of epidermal thickness in 3D skin equivalents treated with DMSO or Etomoxir for 7 days. Measurements were taken from 9–10 randomly selected high-power fields (hpf) per sample. n = 6. Data are presented as mean ± SD. *p*-values were calculated using a paired two-tailed *t*-test. (J) Quantification of retained nuclei per hpf in 3D skin equivalents treated with DMSO or Etomoxir for 7 days. Nuclei were counted blindly across 9–10 randomly selected hpf per sample. n = 6. Data are presented as mean ± SD. *p*-values were calculated using a paired two-tailed *t*-test. (K) Schematic of the mouse model. (L) Western blot analysis of CPT1A expression in the epidermis of wild-type (CPT1A^f/f^) and K14Cre; CPT1A^f/f^ mice at postnatal day 3 (P3). Vinculin was used as a loading control. (M) Representative H&E-stained images of skin from wild-type (CPT1A^f/f^) and K14Cre; CPT1A^f/f^ mice at 7 weeks of age, with or without tape stripping. Scale bar, 100 μm. (N) Quantification of basal layer thickness in wi ld-type (CPT1A^f/f^) and K14Cre; CPT1A^f/f^ mouse skin at 7 weeks, with or without tape stripping (TP). Basal layer was visualized using KRT5 immunostaining and measured in 9–10 randomly selected hpf per sample. n ≤ 4. Data are presented as mean ± SD. *p*-values were calculated using an unpaired two-tailed *t*-test.

**Figure 4:**
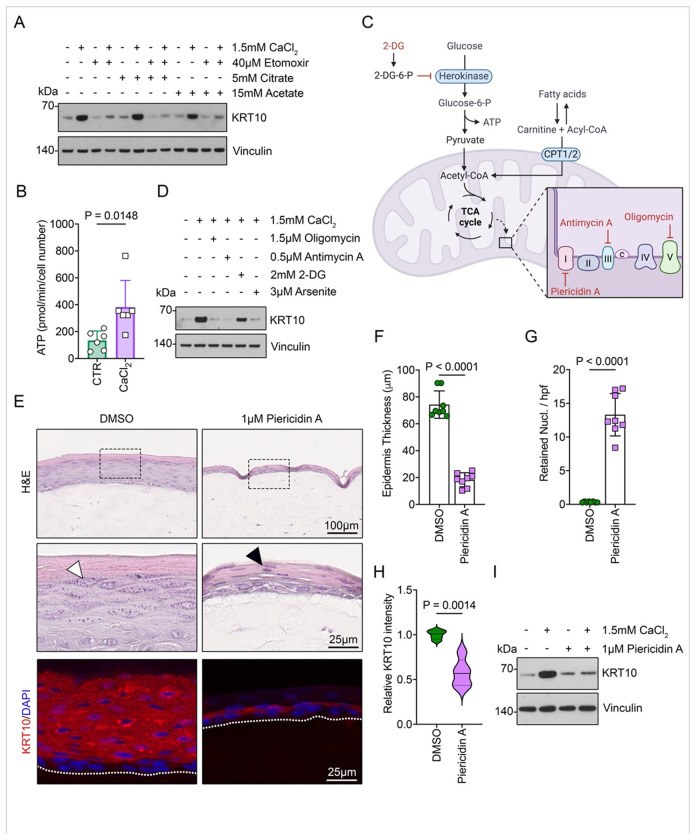
ATP production sustained by FAO is crucial for epidermal homeostasis. (A) Western blot analysis of KRT10 expression in ESC treated with DMSO, Etomoxir, citrate, or acetate for 24 hours under basal or differentiated conditions. Vinculin was used as a loading control. (B) ATP production in ESC under basal or differentiated conditions. Cells were sequentially treated with oligomycin, FCCP, and rotenone/antimycin A as indicated. n = 6 donors. Data are presented as mean ± SD. *p*-values were determined using a paired two-tailed *t*-test. (C) Schematic illustration of the relevant metabolic pathways and respective treatments. (D) Western blot analysis of KRT10 expression in ESC treated with DMSO, oligomycin, antimycin A, 2-DG, or arsenite for 24 hours under basal or differentiated conditions. Vinculin was used as a loading control. (E) Representative low- and high-magnification images of hematoxylin and eosin (H&E) staining and immunofluorescence (IF) for KRT10 in 3D skin cultures treated with DMSO or Piericidin A for 7 days. White arrowheads indicate keratohyalin granules; black arrowheads indicate retained nuclei in the cornified layers. Dashed lines mark the bottom of the epidermis. Scale bars: black, 100 μm (low magnification) and 25 μm (high magnification); white, 25 μm. (F) Quantification of epidermal thickness in 3D skin equivalents treated with DMSO or Piericidin A for 7 days. Measurements were taken from 9–10 randomly selected hpf per culture. n = 8. Data are presented as mean ± SD. *p*-values were determined using a paired two-tailed *t*-test. (G) Quantification of retained nuclei per hpf in 3D skin equivalents treated with DMSO or Piericidin A for 7 days. Nuclei were blindly counted across 9–10 randomly selected hpf per culture. n = 6. Data are presented as mean ± SD. *p*-values were determined using a paired two-tailed *t*-test. (H) Quantification of relative KRT10 intensity in 3D skin equivalents treated with DMSO or Piericidin A for 7 days. Fluorescence intensity was measured in 9–10 randomly selected hpf per culture. n = 5. Data are presented as mean ± SD. *p*-values were determined using a paired two-tailed *t*-test. (I) Western blot analysis of KRT10 expression in ESC treated with DMSO or Piericidin A for 24 hours under basal or differentiated conditions. Vinculin was used as a loading control

## References

[1] Slominski A. & Pawelek J. Animals under the sun: effects of ultraviolet radiation on mammalian skin. Clin Dermatol 16, 503–515 (1998). 9699062 10.1016/s0738-081x(98)00023-6

[2] Fuchs E. & Nowak J.A. Building epithelial tissues from skin stem cells. Cold Spring Harb Symp Quant Biol 73, 333–350 (2008). 19022769 10.1101/sqb.2008.73.032 PMC2693088

[3] Fuchs E. Skin stem cells: rising to the surface. J Cell Biol 180, 273–284 (2008). 18209104 10.1083/jcb.200708185 PMC2213592

[4] Ellis S.J., Distinct modes of cell competition shape mammalian tissue morphogenesis. Nature 569, 497–502 (2019). 31092920 10.1038/s41586-019-1199-y PMC6638572

[5] Jensen K.B. & Watt F.M. Single-cell expression profiling of human epidermal stem and transit-amplifying cells: Lrig1 is a regulator of stem cell quiescence. Proc Natl Acad Sci U S A 103, 11958–11963 (2006). 16877544 10.1073/pnas.0601886103 PMC1567680

[6] Rompolas P., Spatiotemporal coordination of stem cell commitment during epidermal homeostasis. Science 352, 1471–1474 (2016). 27229141 10.1126/science.aaf7012 PMC4958018

[7] Mesa K.R., Homeostatic Epidermal Stem Cell Self-Renewal Is Driven by Local Differentiation. Cell Stem Cell 23, 677–686 e674 (2018). 30269903 10.1016/j.stem.2018.09.005 PMC6214709

[8] Cockburn K., Gradual differentiation uncoupled from cell cycle exit generates heterogeneity in the epidermal stem cell layer. Nat Cell Biol 24, 1692–1700 (2022). 36357619 10.1038/s41556-022-01021-8 PMC9729105

[9] Elsholz F., Harteneck C., Muller W. & Friedland K. Calcium--a central regulator of keratinocyte differentiation in health and disease. Eur J Dermatol 24, 650–661 (2014). 25514792 10.1684/ejd.2014.2452

[10] Blanpain C. & Fuchs E. p63: revving up epithelial stem-cell potential. Nat Cell Biol 9, 731–733 (2007). 17603506 10.1038/ncb0707-731

[11] Koster M.I., p63 induces key target genes required for epidermal morphogenesis. Proc Natl Acad Sci U S A 104, 3255–3260 (2007). 17360634 10.1073/pnas.0611376104 PMC1805532

[12] Nguyen B.C., Cross-regulation between Notch and p63 in keratinocyte commitment to differentiation. Genes Dev 20, 1028–1042 (2006). 16618808 10.1101/gad.1406006 PMC1472299

[13] Kolev V., EGFR signalling as a negative regulator of Notch1 gene transcription and function in proliferating keratinocytes and cancer. Nat Cell Biol 10, 902–911 (2008). 18604200 10.1038/ncb1750 PMC2747621

[14] Mandinova A., The FoxO3a gene is a key negative target of canonical Notch signalling in the keratinocyte UVB response. EMBO J 27, 1243–1254 (2008). 18388864 10.1038/emboj.2008.45 PMC2367396

[15] Nicolas M., Notch1 functions as a tumor suppressor in mouse skin. Nat Genet 33, 416–421 (2003). 12590261 10.1038/ng1099

[16] Okuyama R., High commitment of embryonic keratinocytes to terminal differentiation through a Notch1-caspase 3 regulatory mechanism. Dev Cell 6, 551–562 (2004). 15068794 10.1016/s1534-5807(04)00098-x

[17] Gangnuss S., Regulation of MAPK activation, AP-1 transcription factor expression and keratinocyte differentiation in wounded fetal skin. J Invest Dermatol 122, 791–804 (2004). 15086567 10.1111/j.0022-202X.2004.22319.x

[18] Mehic D., Bakiri L., Ghannadan M., Wagner E.F. & Tschachler E. Fos and jun proteins are specifically expressed during differentiation of human keratinocytes. J Invest Dermatol 124, 212–220 (2005). 15654976 10.1111/j.0022-202X.2004.23558.x

[19] Han B., Suppression of AP1 transcription factor function in keratinocyte suppresses differentiation. PLoS One 7, e36941 (2012). 22649503 10.1371/journal.pone.0036941 PMC3359321

[20] Wang X., AP-2alpha: a regulator of EGF receptor signaling and proliferation in skin epidermis. J Cell Biol 172, 409–421 (2006). 16449191 10.1083/jcb.200510002 PMC2063650

[21] Watt F.M. Stem cell fate and patterning in mammalian epidermis. Curr Opin Genet Dev 11, 410–417 (2001). 11448627 10.1016/s0959-437x(00)00211-2

[22] Watt F.M. Epidermal stem cells: markers, patterning and the control of stem cell fate. Philos Trans R Soc Lond B Biol Sci 353, 831–837 (1998). 9684280 10.1098/rstb.1998.0247 PMC1692275

[23] Koster M.I. & Roop D.R. Genetic pathways required for epidermal morphogenesis. Eur J Cell Biol 83, 625–629 (2004). 15679107 10.1078/0171-9335-00387

[24] Koster M.I., Huntzinger K.A. & Roop D.R. Epidermal differentiation: transgenic/knockout mouse models reveal genes involved in stem cell fate decisions and commitment to differentiation. J Investig Dermatol Symp Proc 7, 41–45 (2002). 10.1046/j.1523-1747.2002.19639.x 12518791

[25] Livshits G., Kobielak A. & Fuchs E. Governing epidermal homeostasis by coupling cell-cell adhesion to integrin and growth factor signaling, proliferation, and apoptosis. Proc Natl Acad Sci U S A 109, 4886–4891 (2012). 22411810 10.1073/pnas.1202120109 PMC3324018

[26] Sen G.L., Webster D.E., Barragan D.I., Chang H.Y. & Khavari P.A. Control of differentiation in a self-renewing mammalian tissue by the histone demethylase JMJD3. Genes Dev 22, 1865–1870 (2008). 18628393 10.1101/gad.1673508 PMC2492733

[27] Sen G.L., Reuter J.A., Webster D.E., Zhu L. & Khavari P.A. DNMT1 maintains progenitor function in self-renewing somatic tissue. Nature 463, 563–567 (2010). 20081831 10.1038/nature08683 PMC3050546

[28] Ezhkova E., Ezh2 orchestrates gene expression for the stepwise differentiation of tissue-specific stem cells. Cell 136, 1122–1135 (2009). 19303854 10.1016/j.cell.2008.12.043 PMC2716120

[29] Flora P. & Ezhkova E. Regulatory mechanisms governing epidermal stem cell function during development and homeostasis. Development 147(2020). 10.1242/dev.194100 PMC7687856 33191273

[30] LeBoeuf M., Hdac1 and Hdac2 act redundantly to control p63 and p53 functions in epidermal progenitor cells. Dev Cell 19, 807–818 (2010). 21093383 10.1016/j.devcel.2010.10.015 PMC3003338

[31] Yi R., Poy M.N., Stoffel M. & Fuchs E. A skin microRNA promotes differentiation by repressing ‘sternness’. Nature 452, 225–229 (2008). 18311128 10.1038/nature06642 PMC4346711

[32] Kwon E., The RNA-binding protein YBX1 regulates epidermal progenitors at a posttranscriptional level. Nat Commun 9, 1734 (2018). 29712925 10.1038/s41467-018-04092-0 PMC5928080

[33] Wang Y., Arribas-Layton M., Chen Y., Lykke-Andersen J. & Sen G.L. DDX6 Orchestrates Mammalian Progenitor Function through the mRNA Degradation and Translation Pathways. Mol Cell 60, 118–130 (2015). 26412305 10.1016/j.molcel.2015.08.014 PMC4592480

[34] Randhawa M., Sangar V., Tucker-Samaras S. & Southall M. Metabolic signature of sun exposed skin suggests catabolic pathway overweighs anabolic pathway. PLoS One 9, e90367 (2014). 24603693 10.1371/journal.pone.0090367 PMC3946127

[35] Hamanaka R.B., Mitochondrial reactive oxygen species promote epidermal differentiation and hair follicle development. Sci Signal 6, ra8 (2013). 23386745 10.1126/scisignal.2003638 PMC4017376

[36] Hamanaka R.B., Weinberg S.E., Reczek C.R. & Chandel N.S. The Mitochondrial Respiratory Chain Is Required for Organismal Adaptation to Hypoxia. Cell Rep 15, 451–459 (2016). 27068470 10.1016/j.celrep.2016.03.044 PMC4838509

[37] Roshan A., Human keratinocytes have two interconvertible modes of proliferation. Nat Cell Biol 18, 145–156 (2016). 26641719 10.1038/ncb3282 PMC4872834

[38] Murai K., Epidermal Tissue Adapts to Restrain Progenitors Carrying Clonal p53 Mutations. Cell Stem Cell 23, 687–699 e688 (2018). 30269904 10.1016/j.stem.2018.08.017 PMC6224607

[39] Singh K., JunB defines functional and structural integrity of the epidermo-pilosebaceous unit in the skin. Nat Commun 9, 3425 (2018). 30143626 10.1038/s41467-018-05726-z PMC6109099

[40] Sheth A.I., Targeting Acute Myeloid Leukemia Stem Cells through Perturbation of Mitochondrial Calcium. Cancer Discov 14, 1922–1939 (2024). 38787341 10.1158/2159-8290.CD-23-1145 PMC11452272

[41] Legros F., Lombes A., Frachon P. & Rojo M. Mitochondrial fusion in human cells is efficient, requires the inner membrane potential, and is mediated by mitofusins. Mol Biol Cell 13, 4343–4354 (2002). 12475957 10.1091/mbc.E02-06-0330 PMC138638

[42] Westermann B. Bioenergetic role of mitochondrial fusion and fission. Biochim Biophys Acta 1817, 1833–1838 (2012). 22409868 10.1016/j.bbabio.2012.02.033

[43] Mellem D., Fragmentation of the mitochondrial network in skin in vivo. PLoS One 12, e0174469 (2017). 28644888 10.1371/journal.pone.0174469 PMC5482427

[44] Simpson C.L., NIX initiates mitochondrial fragmentation via DRP1 to drive epidermal differentiation. Cell Rep 34, 108689 (2021). 33535046 10.1016/j.celrep.2021.108689 PMC7888979

[45] Jang C., Metabolite Exchange between Mammalian Organs Quantified in Pigs. Cell Metab 30, 594–606 e593 (2019). 31257152 10.1016/j.cmet.2019.06.002 PMC6726553

[46] Vacanti N.M., Regulation of substrate utilization by the mitochondrial pyruvate carrier. Mol Cell 56, 425–435 (2014). 25458843 10.1016/j.molcel.2014.09.024 PMC4267523

[47] Halestrap A.P. & Denton R.M. The specificity and metabolic implications of the inhibition of pyruvate transport in isolated mitochondria and intact tissue preparations by alpha-Cyano-4-hydroxycinnamate and related compounds. Biochem J 148, 97–106 (1975). 1171687 10.1042/bj1480097 PMC1165510

[48] Kruszynska Y.T. & Sherratt H.S. Glucose kinetics during acute and chronic treatment of rats with 2[6(4-chloro-phenoxy)hexyl]oxirane-2-carboxylate, etomoxir. Biochem Pharmacol 36, 3917–3921 (1987). 3689429 10.1016/0006-2952(87)90458-8

[49] Yao C.H., Identifying off-target effects of etomoxir reveals that carnitine palmitoyltransferase I is essential for cancer cell proliferation independent of beta-oxidation. PLoS Biol 16, e2003782 (2018). 29596410 10.1371/journal.pbio.2003782 PMC5892939

[50] Schoors S., Fatty acid carbon is essential for dNTP synthesis in endothelial cells. Nature 520, 192–197 (2015). 25830893 10.1038/nature14362 PMC4413024

[51] Potten C.S., Proliferation in murine epidermis after minor mechanical stimulation. Part 1. Sustained increase in keratinocyte production and migration. Cell Prolif 33, 231–246 (2000). 11041204 10.1046/j.1365-2184.2000.00178.x PMC6496670

[52] Pinkus H. Examination of the epidermis by the strip method of removing horny layers. I. Observations on thickness of the horny layer, and on mitotic activity after stripping. J Invest Dermatol 16, 383–386 (1951). 14841399 10.1038/jid.1951.45

[53] Zhang P., FBP1 orchestrates keratinocyte proliferation/differentiation and suppresses psoriasis through metabolic control of histone acetylation. Cell Death Dis 15, 392 (2024). 38834617 10.1038/s41419-024-06706-6 PMC11150480

[54] Wellen K.E., ATP-citrate lyase links cellular metabolism to histone acetylation. Science 324, 1076–1080 (2009). 19461003 10.1126/science.1164097 PMC2746744

[55] Zheng X., Alleviation of neuronal energy deficiency by mTOR inhibition as a treatment for mitochondria-related neurodegeneration. Elife 5(2016). 10.7554/eLife.13378 PMC4846388 27008180

[56] Sabir S., Role of cadmium and arsenic as endocrine disruptors in the metabolism of carbohydrates: Inserting the association into perspectives. Biomed Pharmacother 114, 108802 (2019). 30921704 10.1016/j.biopha.2019.108802

[57] Stein L.R. & Imai S. The dynamic regulation of NAD metabolism in mitochondria. Trends Endocrinol Metab 23, 420–428 (2012). 22819213 10.1016/j.tem.2012.06.005 PMC3683958

[58] Bigas J., Sevilla L.M., Carceller E., Boix J. & Perez P. Epidermal glucocorticoid and mineralocorticoid receptors act cooperatively to regulate epidermal development and counteract skin inflammation. Cell Death Dis 9, 588 (2018). 29789551 10.1038/s41419-018-0673-z PMC5964110

[59] Zhou X., Chen Y., Cui L., Shi Y. & Guo C. Advances in the pathogenesis of psoriasis: from keratinocyte perspective. Cell Death Dis 13, 81 (2022). 35075118 10.1038/s41419-022-04523-3 PMC8786887

[60] Schmuth M., Skin Barrier in Atopic Dermatitis. J Invest Dermatol 144, 989–1000 e1001 (2024). 38643989 10.1016/j.jid.2024.03.006

[61] Leman G., Mitochondrial Activity Is Upregulated in Nonlesional Atopic Dermatitis and Amenable to Therapeutic Intervention. J Invest Dermatol 142, 2623–2634 e2612 (2022). 35341734 10.1016/j.jid.2022.01.035

